# Operator-related low diagnostic quality in bitewing examinations performed with sensors

**DOI:** 10.2340/aos.v85.46001

**Published:** 2026-05-07

**Authors:** Daniel Olsson, Eva Levring Jäghagen, Maria Garoff

**Affiliations:** Oral and Maxillofacial Radiology, Department of Odontology, Umeå University, Sweden

**Keywords:** Radiography, bitewing, dentistry, radiography, dental, digital, diagnostic imaging

## Abstract

**Objective:**

Bitewing radiographs are essential for caries and marginal bone diagnostics. Diagnostic quality depends on operator technique. This study evaluated patient-level diagnostic quality of sensor-based bitewing examinations to identify operator-related deficiencies.

**Material and Methods:**

In this retrospective, cross-sectional study, 962 bitewing examinations from 31 Swedish public dental practices were randomly selected and evaluated for caries and marginal bone level diagnostic image quality, according to European guidelines. Available panoramic/periapical images acquired in connection with the bitewing examinations were included in a second quality assessment. Recorded deficiencies included sensor placement, collimation artifacts, and insufficient biting on the sensor holder. Associations between diagnostic quality and sensor size, number of images, age group, jaw, side, and sex were analyzed using χ^2^ test and logistic regression. Three calibrated examiners performed the evaluations.

**Results:**

The requirements were fulfilled in 5% and acceptable in 43% of the bitewing examinations, increasing to 7% and 45% when including panoramic/periapical images. Quality was better with larger sensor sizes (*p* < 0.001), more exposures (*p* < 0.001), panoramic/periapical images (*p* < 0.001), age ≥12 years (*p* < 0.001), and in the maxilla (*p* < 0.001). Common errors were incorrect sensor placement (94%), collimation artifacts (57%), and insufficient biting (15%). No differences were found between side or sex. Inter-observer agreement was substantial (Fleiss’ kappa = 0.61; Gwet’s AC1 = 0.62); intra-observer agreement was almost perfect (Cohen’s kappa = 0.88).

**Conclusions:**

Most bitewing examinations, especially in children, fail to meet diagnostic requirements due to deficient operator performance and quality assessment. Panoramic/periapical images may improve diagnostic quality but should not replace optimized bitewing examinations. Targeted continuing education is required.

## Introduction

Bitewings are the most commonly performed dental radiographic examinations in general dentistry, allowing for the assessment of interproximal caries and marginal bone levels that are not visible through clinical inspection [1–4]. Bitewings are usually obtained during routine dental examinations and enable the evaluation of disease progression and treatment efficacy [1–3, 5].

To ensure accurate caries and marginal bone level diagnostics, bitewing examinations must meet specific quality requirements. Although these requirements may vary between countries, the overarching guidelines from the European Union state that, ideally, there should be no horizontal overlap between adjacent teeth from the distal surfaces of the canines to the mesial surfaces of the most posterior erupted teeth. If overlap is present, it should not obscure more than half the enamel thickness to maintain diagnostic value. In addition, the images should depict marginal bone levels in both the maxilla and mandible, enabling assessment of potential marginal bone loss [6].

Previous studies that evaluated the diagnostic quality of analogue films found that only 55–59% of bitewings were deemed satisfactory [7–9]. However, knowledge regarding the diagnostic quality of bitewing examinations obtained using digital techniques is lacking. Digital sensors (charge-coupled device [CCD] and complementary metal-oxide-semiconductor [CMOS] sensors), when compared to film, are thick, rigid and non-adaptable to facilitate placement and thereby cause more discomfort for the patient and impose higher demands on the operators’ performance [10]. These factors can complicate sensor placement and may compromise diagnostic quality, particularly in children [11, 12]. Compared to digital sensors, digital photostimulable phosphor systems (PSP) are easier to position due to their thinner and more flexible design, often resulting in lower patient discomfort and fewer retakes. PSP systems also offer a larger capture area than sensors, enabling more comprehensive visualization of the region of interest [13, 14]. Disadvantages of PSP include a lack of immediate feedback and risk of artifacts during scanning [10].

Bitewing examinations are performed by dental professionals, and in Sweden, the examinations must always be justified and approved by authorized professionals. To meet diagnostic quality requirements, operators must possess adequate expertise in the imaging technique. The most prevalent deficiencies related to the imaging technique for bitewings include improper sensor placement, incorrect horizontal beam angulation, and collimation artifacts [7–9, 15–17]. Previous studies have primarily reported the quality of single radiographic images in adults prior to procedures, such as prosthodontic treatment. Bitewing examinations at the patient level have not been assessed for overall quality regarding the European Union’s requirements for caries and marginal bone level diagnostics [6]. When digital techniques were first introduced, a study only including adults showed a slight increase in deficiencies compared to film [17], and operators found it difficult to position digital sensors correctly [13]. Considering the current widespread use of digital systems with sensors, operator-related factors influencing the overall diagnostic quality of digital bitewing examinations need to be explored across all age groups.

This study aimed to i) evaluate the overall quality of digital bitewing examinations at the patient level regarding caries and marginal bone level diagnostics and ii) to identify operator-related deficiencies. We also assessed if panoramic and/or periapical examinations contribute to the diagnostics.

## Materials and methods

### Study participants

**Figure 1:**
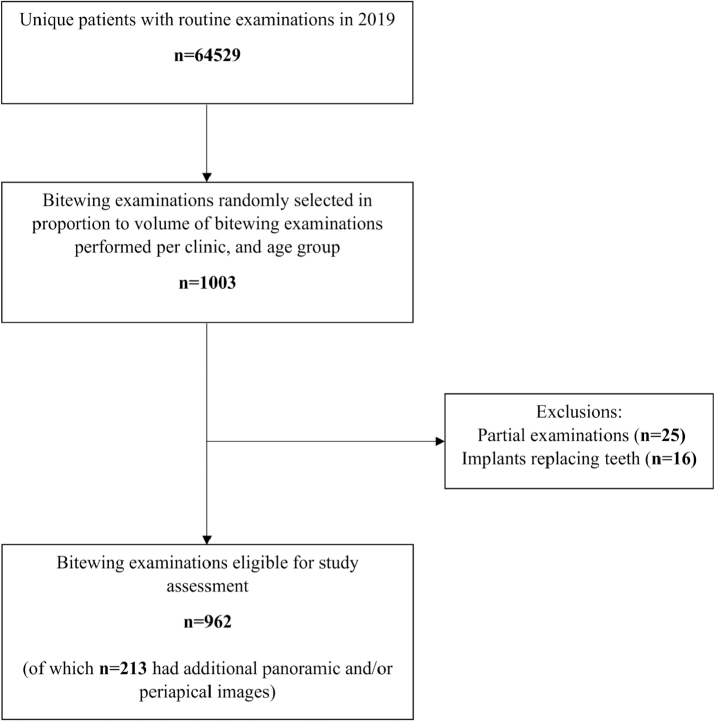
Flowchart of the selection of bitewing examinations.

Participants in this retrospective cross-sectional study were randomly selected from all patients who underwent routine examinations at any of the 31 public general dental practices in Region Västerbotten, Sweden, in 2019. A sample size of 1,000 patients was pragmatically deemed sufficient for the intended analyses. To ensure representativeness, the selection was stratified so that each clinic contributed a volume of cases in proportion to its total annual examination volume. The sample was further selected in proportion to patient age groups (< 8, 8–12, 13–17, 18–39, 40–59, 60–79, and ≥ 80 years) to reflect the age distribution of patients who underwent routine bitewing examinations (Figure 1). Inclusion criteria were bilateral bitewing examinations depicting both the upper and lower jaws. Patients with implants at the level of canines and posteriorly were excluded. In total, 962 bitewing examinations were included in the final study analysis. The patient’s sex, age, date of clinical routine examination, including the bitewing examination, sensor size, and total number of exposed bitewing images, were recorded.

The Swedish Ethical Review Authority approved this study (Dnr. 2021-03456), and written informed consent was not required. This study adheres to Strengthening the Reporting of Observational Studies in Epidemiology (STROBE) guidelines.

### Radiographic examinations

All bitewing examinations (*n* = 962), conducted within 1 month before or after the date of the routine clinical examination, were reviewed after retrieving them from the participants’ dental records (T4 Carestream Dental/USA^®^; Onepix Unident/Sweden^®^). All bitewing images for each examination were included. Panoramic and periapical images taken during the same visit or in close proximity were also retrieved and included in a second assessment (*n* = 213 examinations). The panoramic images were independent examinations, not complementary to compensate for deficiencies in the bitewing examinations. Periapical images were either independent or complementary.

The radiographic examinations were performed by dental professionals using corded CMOS sensors (Schick 33, Dentsply Sirona/USA^®^) and collimation for sensor size 2. Three sensor sizes were used with the following capture area: small 18 × 24 mm, medium 20 × 30 mm, large 25.6 × 36 mm (outer dimensions height × width × thickness; small 23.6 × 31.9 × 7.5 mm, medium 25.4 × 38.3 × 7.5 mm, large 31.2 × 43.0 × 7.5 mm). A bitewing sensor holder with an aiming rod was used almost exclusively (Green Line SnapBite Unident/Sweden^®^).

### Calibration, inter- and intra-observer agreement

Prior to the study assessments, calibration on how to assess the diagnostic quality criteria was performed by two oral and maxillofacial radiologists (OMR) (E.L.J. and M.G.) and a general dentist (D.O.) with analyses of 289 examinations that were not included in the study sample.

All three observers independently assessed 242 (25%) of the 962 bitewing examinations included in the study. All bitewing examinations were evaluated for overall diagnostic quality at the patient level. When available and when deficiencies were identified in the bitewing examination, panoramic/periapical images were considered in a second assessment. Inter-observer agreement was assessed for a subset of the examinations. The remaining study assessments were conducted by one primary observer (D.O.). The performance of the primary observer was further evaluated, and intra-observer agreement was assessed by repeated evaluation of 50 randomly selected examinations performed more than 18 months after the initial assessment.

### Diagnostic quality requirements for bitewing examinations

Diagnostic quality was evaluated following requirements of the European Union [6], formulated by an expert group in OMR, describing obvious radiographic criteria that should be fulfilled. The guidelines align with the requirements for bitewing examinations in public dental practices in Region Västerbotten. For caries diagnostics, the distal surfaces of the canines to the most distal interproximal surfaces had to be depicted. Horizontal overlaps of up to half the enamel thickness were considered to meet the study requirements, whereas restorative junctions had to be free from overlap. Horizontal overlap was included in the overall diagnostic quality assessment but not registered separately. For marginal bone level diagnostics, the marginal bone distal to the canines to the distal surfaces of the most posterior erupted teeth had to be depicted.

### Assessment of bitewing examinations

Overall diagnostic quality at the patient level was assessed in two steps: (1) Bitewings‑only and (2) bitewing examinations including additional panoramic/periapical images, if available. To enable comparisons between jaws and jaw sides, bitewing examinations were also assessed at the quadrant level, strictly adhering to the predefined quality guidelines and using the same two assessment steps. The overall diagnostic quality assessment considered the radiographic dental status and the hypothetical number of additional bitewings needed to fulfill the requirements, regardless of retakes or complementary images. The number of already existing radiographs was not considered since the key factor was whether the areas of interest were missing or inadequately depicted.

**Figure 2:**
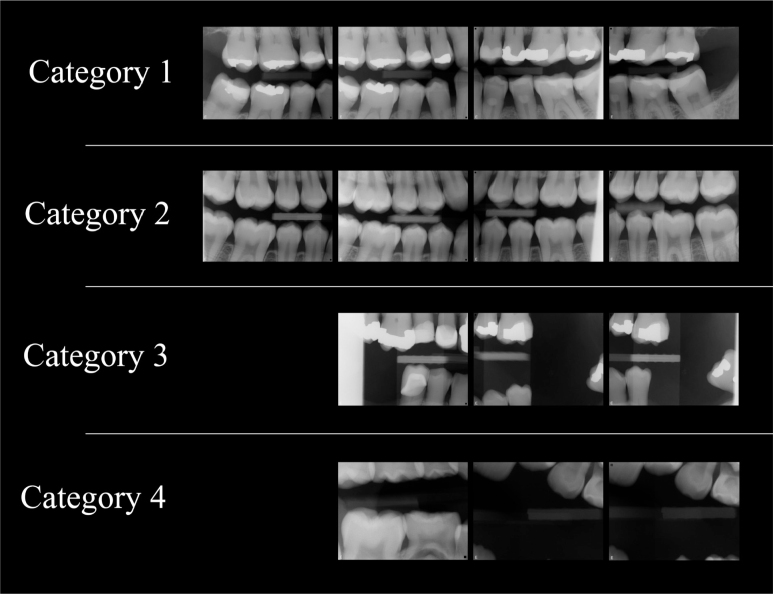
Examples of bitewing examinations of different categories based on how well they fulfilled the quality requirements for caries and marginal bone level diagnostics. Category 1: All required interproximal surfaces depicted with minimal or no overlap, including all required areas of the marginal bone. Category 2: Marginal bone levels insufficiently depicted posterior to the most posterior teeth in all required areas, but otherwise healthy radiographic dental conditions. Category 3: Marginal bone levels insufficiently depicted in quadrants 1, 2, and 3, interproximal surfaces are missing or overlapped, including insufficient biting on the sensor holder. Category 4: Requirements not fulfilled and complete retake needed.

The overall diagnostic quality assessment was classified into four categories [18]: category 1 (excellent), no retakes or complementary images needed since all diagnostic requirements for caries and marginal bone level diagnostics were fully met; category 2, (diagnostically acceptable) one or two estimated additional retakes or complementary images needed. Applied when nearly all requirements were fulfilled, with only minor deficiencies such as one or two overlaps greater than half the enamel thickness, one or two interproximal surfaces not depicted or one or two interproximal marginal bone levels not visible, provided that the remaining depicted areas were diagnostically adequate or the overall radiographic conditions appeared healthy; category 3 (diagnostically compromised), two to four estimated additional retakes or complementary images needed. Assigned when several requirements were not fulfilled. These cases had multiple quadrants with missing marginal bone levels and interproximal surface depiction, or several overlaps of more than half of the enamel thickness, reducing the value of caries or bone level diagnostics, although some diagnostic value remained; category 4 (unacceptable), complete retake needed. Referring to examinations where the diagnostic requirements were not fulfilled at all. Deficiencies consisted of widespread overlaps greater than half of the enamel thickness, large areas not depicted, or missing marginal bone levels in multiple sites. Neither caries nor marginal bone level diagnostics could be reliably performed (Figure 2).

### Data treatment

The categories of overall diagnostic quality were dichotomized and assessed in both steps (bitewings‑only and bitewing examinations, including available additional images), where the examinations in categories 1 and 2 were considered to be of acceptable quality and those in categories 3 and 4 were considered to be of unacceptable quality. It was separately recorded if the additional images complemented the assessment of marginal bone levels, caries, or both. The overall diagnostic quality evaluation, including both assessment steps, was divided into age groups: < 8, 8–11, < 12, 12–17, 18–79, and ≥ 80 years. The age groups were defined to reflect developmental stages across life’s early years [19], to facilitate comparison between primary-to-late mixed dentition and permanent dentition, and to assess the influence of dental status or other age-related factors relevant for image quality. Dichotomized data were compared with sensor size, age group, patient sex, presence, and complementary function of additional panoramic/periapical images, and number of exposed bitewings.

Differences in quality for caries and marginal bone level diagnostics between jaws and jaw sides were assessed using quadrant analyses.

Deficiencies in sensor placement were recorded when the most anterior and/or posterior areas were depicted inadequately. Images containing collimation artifacts and insufficient biting on the sensor holder were recorded and assessed for their impact on diagnostic quality. Insufficient biting was defined as failure to bite properly on the sensor holder, resulting in large interocclusal distance and incomplete depiction of teeth and/or marginal bone levels.

### Statistical analysis

Inter-observer agreement for overall diagnostic quality was analyzed using Fleiss’ kappa [20] and Gwet’s AC1 [21]. Observer performance was further analyzed using a Dawid-Skene model [22]. Intra-observer agreement was analyzed using Cohen’s kappa [23]. Differences in paired binary outcomes between the bitewings-only assessment and the assessment including additional images were analyzed using McNemar’s test among the 213 patients with additional images. Comparisons of categorical variables were performed using the two-sided χ^2^ test. The association between diagnostic quality and the number of exposed bitewing images was analyzed with binary logistic regression. *P* < 0.05 was considered significant. All statistical analyses were performed using R Statistical Software (v.4.4.2). Cohen’s kappa, Fleiss’ kappa, and bootstrapped CIs were analyzed using the irr package and bootBCa function, Gwet’s AC1 was analyzed using the rel package, and the CRAN rater package [24] (1.3.1) was used for the Dawid-Skene model.

## Results

In total, 64,529 unique patients had routine examinations conducted in 2019 at public general dental practices in Region Västerbotten, Sweden, and of these, 1,003 examinations were randomly selected for inclusion in the study. Another 289 examinations were randomly selected for the observers’ calibration. Among the 1,003 examinations, 25 were excluded due to incomplete examinations and 16 due to the presence of dental implants, resulting in 962 examinations included for the final analysis (Figure 1). Of the included examinations, 445 (46%) were conducted on males and 517 (54%) on females. Patient age ranged from 4 to 96 years (mean age: 32.9 ± 27.9 years). The distribution across age groups was: <8 years 160 (17%), 8–11 years 119 (12%), 12–17 years 212 (22%), 18–79 years 359 (37%), and ≥ 80 years 112 (12%). In 213 (22%) of the bitewing examinations, additional panoramic and/or periapical images were available.

Both inter-observer analyses indicated substantial agreement between observers (Fleiss’ kappa 0.61 95% confidence interval [CI]: 0.53–0.68 and Gwet’s AC1 (0.62 95% CI: 0.54–0.7)) [20, 21, 25]. The Dawid-Skene model [22] estimated the primary reviewer’s (D.O.) accuracy to be 0.90 (standard error [SE] = 0.024), which was comparable to or higher than that of the two OMRs (0.84 ± 0.027 and 0.86 ± 0.025, respectively). Intra-observer agreement was almost perfect [23], when the primary reviewer (D.O.) repeated assessments of 50 examinations (Cohen’s kappa 0.88, 95% CI: 0.72–1.00).

### Overall assessment of bitewings-only

All requirements were fulfilled (category 1) in 51 (5%), and nearly all requirements were fulfilled (category 2) in 359 (37%) of the examinations. Several requirements were not fulfilled (category 3) in 522 (54%) of the examinations, and no requirements were fulfilled (category 4) in 30 (3%) of examinations. After dichotomization, 410 (43%) of the examinations were assessed to have acceptable quality, and 552 (57%) were assessed to have unacceptable quality.

Analyses using dichotomized data revealed that the overall diagnostic quality was better with larger sensor size (*p* < 0.001). Of the 667 examinations conducted with a large sensor, 332 (50%) had acceptable quality, compared with 46 of 160 (29%) with a medium sensor size, 29 of 127 (23%) with a small sensor size, and three of eight (38%) with mixed sensor sizes (Table 1). Overall diagnostic quality was more frequently acceptable in examinations that included a greater number of bitewing radiographs (2–12; *p* < 0.001).

**Table 1:** Comparison of overall diagnostic quality in bitewing examinations using both the bitewings-only and the additional assessment, including panoramic and/or periapical images when available, distributed on sensor sizes, age groups, and patient sex.

Assessment step		Bitewings-only			Bitewing examination, including panoramic and/or periapical images when available		
Overall diagnostic quality		Acceptable assessment	Unacceptable assessment	*P* *	Acceptable additional assessment	Unacceptable additional assessment	*P* *
**Sensor size used for bitewing imaging** ^**a**^	Large	332 (50)	335 (50)	**< 0.001**	-	-	**-**
	Medium	46 (29)	114 (71)		-	-	
	Small	29 (23)	98 (77)		-	-	
**Age**	< 8 years	36 (23)	124 (78)	**< 0.001**	39 (24)	121 (76)	**< 0.001**
	≥ 8 years	374 (47)	428 (53)		396 (49)	406 (51)	
	8–11 years	37 (31)	82 (69)	**< 0.009**	43 (36)	76 (64)	**0.033**
	≠ 8–11 years	373 (44)	470 (56)		392 (46)	451 (54)	
	12–17 years	130 (61)	82 (39)	**< 0.001**	131 (62)	81 (38)	**< 0.001**
	≠ 12–17 years	280 (37)	470 (63)		304 (41)	446 (59)	
	< 12 years	73 (26)	206 (74)	**< 0.001**	82 (29)	197 (71)	**< 0.001**
	≥ 12 years	337 (49)	346 (51)		353 (52)	330 (48)	
	18–79 years	165 (46)	194 (54)	0.121	174 (48)	185 (52)	0.118
	≠ 18–79 years	245 (41)	358 (59)		261 (43)	342 (57)	
	< 80 years	368 (43)	482 (57)	0.287	387 (46)	463 (54)	0.665
	≥ 80 years	42 (38)	70 (62)		48 (43)	64 (57)	
**Sex**	Male	192 (43)	253 (57)	0.810	208 (46)	237 (54)	0.415
	Female	218 (42)	299 (58)		227 (44)	290 (56)	

Values are given as *n* (%).

a Mixed sensor sizes not included due to small sample size.

* Two-sided χ^2^ test.

Significant values are given in bold.

### Overall assessment of bitewings and additional panoramic and/or periapical images

All requirements were fulfilled (category 1) in 65 (7%) and nearly all requirements were fulfilled (category 2) in 370 (38%) of the examinations. Several requirements were not fulfilled (category 3) in 498 (52%), and no requirements were fulfilled (category 4) in 29 (3%). After dichotomization, 435 (45%) of the examinations were classified as acceptable and 527 (55%) as unacceptable.

### Comparison between bitewings-only and bitewing examinations, including additional panoramic and/or periapical images

Examinations that included additional images were significantly more often assessed as having acceptable quality than the examinations only including bitewing radiographs (120/213 [56%] vs. 315/749 [42%] were acceptable; *p* < 0.001). Compared with the bitewings-only assessment, 25 examinations (12% of the examinations including panoramic and/or periapical images) were reclassified from unacceptable to acceptable when additional images were considered. These reclassifications were due to improved possibility to assess marginal bone level in 13 examinations, caries in two examinations, and both marginal bone level and caries in 10 examinations.

In the bitewings-only assessment, 95 of 213 (45%) examinations with additional images met the criteria for acceptable quality, increasing to 120 (56%) when additional images were considered, reflecting a significant shift in a paired comparison (*p* < 0.001).

The frequency of acceptable quality was significantly higher among participants aged ≥12 years in both assessments (*p* < 0.001), which also corresponds with more frequent use of large sensors. Both assessments demonstrated a significantly lower frequency of acceptable quality in children <8 years (*p* < 0.001) and in those aged 8–11 years (bitewings-only: *p* = 0.009; additional assessment: *p* < 0.033). In contrast, adolescents aged 12–17 years showed a significantly higher frequency of acceptable diagnostic quality compared with all other age groups (bitewings-only: *p* < 0.001; additional assessment: *p* = 0.033).

For adults aged 18–79 years, overall diagnostic quality did not differ significantly from that of any other age group in either assessment (bitewings-only: *p* = 0.121; additional assessment: *p* = 0.118). Similarly, no significant differences were found for the elderly group aged ≥80 years (bitewings-only: *p* = 0.287; additional assessment: *p* = 0.665), nor between male and female patients (bitewings-only: *p* = 0.810; additional assessment: *p* = 0.415) (Table 1).

### Analyses per quadrant and jaw

Based on quadrant analyses, 202 (21%) bitewings-only fulfilled the requirements solely for caries diagnostics, whereas 123 (13%) met the requirements for marginal bone level diagnostics. In the additional assessment, 209 (22%) fulfilled the requirements for caries diagnostics and 146 (15%) for marginal bone level diagnostics. There was no significant difference in fulfilling the requirements between the left and right sides (bitewings-only: *p* = 0.439; additional assessment: *p* = 0.647). However, the requirements were more frequently met in the maxilla than in the mandible, regardless of side, in both assessments (*p* < 0.001). A comparison between jaws revealed that the distal surfaces of the mandibular canines and the maxillary marginal bone level distal to the most posteriorly erupted teeth were less frequently depicted according to both assessments (*p* < 0.001) (Table 2).

**Table 2:** Comparison of the fulfillment of quality requirements in examinations based on subgroups, using both the bitewings-only and the additional assessment, including panoramic and/or periapical images when available.

Assessment step		Bitewings-only			Bitewing examination, including panoramic and/or periapical images when available		
Quality requirement		Fulfilled quality requirements	Did not fulfill quality requirements	*P* *	Fulfilled quality requirements	Did not fulfill quality requirements	*P* *
**Side**	Left	165 (17)	797 (83)	0.439	186 (19)	776 (81)	0.647
	Right	179 (19)	783 (81)		195 (20)	767 (80)	
**Jaw**	Maxilla	235 (24)	727 (76)	**< 0.001**	253 (26)	709 (74)	**< 0.001**
	Mandible	165 (17)	797 (83)		176 (18)	786 (82)	
**Depicted distal surfaces of canines**	Maxilla	517 (54)	445 (46)	**< 0.001**	525 (55)	437 (45)	**< 0.001**
	Mandible	284 (30)	678 (70)		288 (30)	674 (70)	
**Depicted marginal bone distal to the most posterior teeth**	Maxilla	437 (45)	525 (55)	**< 0.001**	470 (49)	492 (51)	**< 0.001**
	Mandible	525 (55)	437 (45)		546 (57)	416 (43)	

Values are given as *n* (%).

* Two-sided χ^2^ test. Significant values are given in bold.

### Common deficiencies

Based on the bitewings-only assessment, the most prevalent deficiency was incorrect sensor placement in anterior–posterior direction, where the sensor was not inserted sufficiently posteriorly and/or anteriorly to include the complete area of interest. This resulted in undepicted marginal bone levels or interproximal tooth surfaces and occurred in 905 (94%) of all examinations. Collimation artifacts were present in 546 (57%) of examinations, and they negatively impacted diagnostic quality in 103 (11%) of the cases. Insufficient biting on the sensor holder compromised diagnostic quality in 147 (15%) of examinations.

## Discussion

The main findings of this study were that more than half of all bitewing examinations performed in public dental practices failed to fulfill the requirements for caries and marginal bone level diagnostics assessed at the patient level. Compromised diagnostic quality was associated with operator-related deficiencies in the imaging technique, particularly incorrect sensor placement. We also clearly demonstrated that larger sensor sizes and greater number of exposed bitewing images significantly improved the diagnostic quality.

To the best of our knowledge, no previous research has explored the diagnostic quality of bitewing examinations including children. We found that bitewing examinations of acceptable quality occurred significantly more often among participants aged ≥12 years. This finding may be confounded by sensor size, as large sensors were more commonly used from 12 years of age onward. This also coincides with the age of early permanent dentition [26], which makes the placement of larger sensors more feasible. It has been shown that sensors cause pain in children [11], and that diagnostic quality can be considerably compromised by the degree of children’s cooperation [27]. In general, digital systems with sensors cause patient discomfort to a greater extent than analog techniques due to their thickness, rigidity, and cord [10]. Still, the use of smaller sensor sizes should be carefully considered, especially in children, due to increased radiation exposure [28, 29] and the risk of compromised diagnostic quality due to the smaller capture area, even when performed using an adequate exposure technique. Digital systems with PSP are generally easier to position, have a larger capture area, and cause less patient discomfort, resulting in fewer retakes [13, 14]. Disadvantages are that there is no immediate feedback on the captured image, the time-consuming need of scanning, and increased risk of artifacts due to the scanning process [10, 30, 31]. However, to our knowledge, no recent study has compared the diagnostic quality of PSP systems with that of sensors at the patient level. Given this study’s results, it can be assumed that the proportion of deficiencies in diagnostic image quality would remain high even with the use of PSP systems, as the issue is often related to the operators’ competence in image acquisition techniques.

When exposing patients to ionizing radiation, the as low as diagnostically acceptable (ALADA) optimization principle should always be applied [28, 32]. Performing, as well as evaluating a bitewing examination without taking other recently acquired radiographic examinations into consideration could result in an underestimation of the diagnostic quality. Panoramic images could aid the assessment of foremost marginal bone levels, while periapical images could contribute to the assessment of marginal bone levels and/or caries when projection geometry was adequate, and their availability may have influenced how the bitewing examinations were performed. Consideration of all existing radiographs is therefore necessary. Our analysis revealed that when panoramic and/or periapical images were available, they significantly increased the number of examinations with acceptable diagnostic quality, from 45% to 56% of the examinations. However, performing additional panoramic and/or periapical images to compensate for deficiencies in bitewing examinations represents an inappropriate approach, as sufficient diagnostic quality of the bitewing examination would eliminate the need for additional images, thereby reducing the patients’ radiation dose, lowering healthcare costs, and aligning with the ALADA principle. When considering the entire study population, the addition of supplementary images resulted in a limited overall improvement in diagnostic quality (2%). This suggests that acquiring additional radiographs solely to compensate for suboptimal bitewing examinations would provide minimal additional diagnostic value. It is particularly important in children to keep the number of acquired radiographic images as low as possible while still adequately addressing the clinical indication, due to their increased radiation sensitivity following higher cell turnover and increased mitotic activity compared to adults [33]. Optimizing diagnostic quality in accordance with the ALADA principle has proven difficult in this study, especially in children <12 years of age.

Our results reveal significantly higher diagnostic quality in the maxilla compared to the mandible. Furthermore, the distal surfaces of the mandibular canines were depicted significantly less often than the maxillary canines, while the marginal bone levels distal to the most posterior teeth in the maxilla were depicted significantly less often than in the mandible. These findings are most likely explained by anatomical differences between the jaws regarding crown shapes and tooth positioning and sometimes by abnormal occlusion, which requires the operator to possess adequate skills in anterior-posterior sensor placement. Given the standard location of the x-ray machine and in relation to the dental unit within the region’s clinics, we hypothesized lower diagnostic quality on the left side compared to the right side; however, our results revealed no significant differences.

The introduction of digital techniques in general dental practice around the turn of the millennium came with the expectation that operator-related deficiencies compromising diagnostic quality would diminish as clinicians adapted to the technology [17]. However, our findings reveal that widespread operator-related deficiencies persist using sensor technology, indicating that compromised diagnostic quality cannot be attributed solely to the detector technology.

A key strength of this study is the large dataset including all age groups and that we evaluated the diagnostic quality of the entire radiographic examination, including both bitewings and additional images, thereby enhancing the relevance for general dental practice. We excluded patients with implants at the level of canines and posteriorly since the requirements to meet diagnostic quality for implants differ from those for bitewing examinations. When compared to teeth, assessment of implants often requires different imaging techniques that can also vary between brands. Further, patients with implants in the defined area were few (*n* = 16), making them non-representative for analysis. It would have been valuable to assess the operators’ experience and educational background, as well as the patient’s ability to cooperate during the examination. However, that information was not available. A limitation of this study is its cross-sectional design, which does not account for present clinical findings or radiographic findings obtained more than 1 month before or after the routine examinations, factors that may have compensated for reduced image quality. There is no predetermined number of radiographs that a bitewing examination should include, but rather as many as required to fulfill the diagnostic requirements for caries and marginal bone level assessment. Although four images are often considered standard, additional exposures may still be required, even with optimal operator technique. Consequently, potential retakes or additional complementary images were neither considered nor identifiable due to the retrospective nature of the study design. It may also be considered a limitation that only one observer performed 75% of the study assessments without a second observer. However, 242 (25%) of the assessments were conducted by all three observers, and the interobserver agreement was substantial according to both Fleiss’ kappa and Gwet’s AC1, which strengthens the reliability of our results. The primary observer was also found to possess high accuracy, according to the Dawid-Skene model and showed almost perfect intra-observer agreement using Cohen’s kappa. In addition, the study assessments followed a very thorough calibration process, which included 289 bitewing examinations and several sessions with discussion to clarify the diagnostic criteria, to ensure that all observers agreed on how to interpret and categorize the diagnostic quality of the examinations. Furthermore, the result on diagnostic quality of the calibration sample was comparable to the study results.

Our results indicate that continuing education strategies are necessary for dental professionals to increase theoretical knowledge of requirements and reflective and practical skills in imaging technique. It is important to assess the diagnostic quality of bitewing examinations while performing them and adjust the imaging technique before retakes. Future studies should examine the impact of continuing education on both the diagnostic quality and operator competence in imaging techniques. The diagnostic quality in various workplace situations should also be compared with the operator’s level of competence in the imaging technique [34, 35].

## Conclusion

The majority of digital bitewing examinations, especially those performed on children, fail to meet the requirements to ensure accurate caries and marginal bone level diagnostics due to deficient operator performance and quality assessment. Independent panoramic and independent or complementary periapical examinations may occasionally compensate for bitewing deficiencies but should not substitute for adequate bitewing quality. Targeted continuing education is required for dental professionals in general dentistry to optimize bitewing imaging and quality assessment.
